# Induced B Cell Development in Adult Mice

**DOI:** 10.3389/fimmu.2018.02483

**Published:** 2018-10-31

**Authors:** Anne-Margarete Brennecke, Sandra Düber, Bishnudeo Roy, Irene Thomsen, Annette I. Garbe, Frank Klawonn, Oliver Pabst, Karsten Kretschmer, Siegfried Weiss

**Affiliations:** ^1^Molecular Immunology, Helmholtz Centre for Infection Research, Braunschweig, Germany; ^2^Medical School Hannover, Institute of Immunology, Hannover, Germany; ^3^Osteoimmunology, DFG-Center for Regenerative Therapies Dresden, Center for Molecular and Cellular Bioengineering, Technische Universität Dresden, Dresden, Germany; ^4^Biostatistics Group, Helmholtz Centre for Infection Research, Braunschweig, Germany; ^5^Institute of Molecular Medicine, RWTH Aachen University, Aachen, Germany; ^6^Molecular and Cellular Immunology/Immune Regulation, DFG-Center for Regenerative Therapies Dresden, Center for Molecular and Cellular Bioengineering, Technische Universität Dresden, Dresden, Germany

**Keywords:** bone marrow, RAG, B cell development, B-2/B-1a/B-1b, antibodies, CSR, T-dependent/-independent, VH usage

## Abstract

We employed the B-Indu-Rag1 model in which the coding exon of recombination-activating gene 1 (Rag1) is inactivated by inversion. It is flanked by inverted loxP sites. Accordingly, B cell development is stopped at the pro/pre B-I cell precursor stage. A B cell-specific Cre recombinase fused to a mutated estrogen receptor allows the induction of RAG1 function and B cell development by application of Tamoxifen. Since Rag1 function is recovered in a non-self-renewing precursor cell, only single waves of development can be induced. Using this system, we could determine that B cells minimally require 5 days to undergo development from pro/preB-I cells to the large and 6 days to the small preB-II cell stage. First immature transitional (T) 1 and T2 B cells could be detected in the bone marrow at day 6 and day 7, respectively, while their appearance in the spleen took one additional day. We also tested a contribution of adult bone marrow to the pool of B-1 cells. Sublethally irradiated syngeneic WT mice were adoptively transferred with bone marrow of B-Indu-Rag1 mice and B cell development was induced after 6 weeks. A significant portion of donor derived B-1 cells could be detected in such adult mice. Finally, early VH gene usage was tested after induction of B cell development. During the earliest time points the VH genes proximal to D/J were found to be predominantly rearranged. At later time points, the large family of the most distal VH prevailed.

## Introduction

B cells are key players in adaptive immunity. However, they also play an important role as innate-like effector cells (1). Various subpopulations can be defined according to these functions. This is also reflected by specific sets of surface markers that are diagnostic for such particular B cell subpopulations. Thus, subpopulations like B-1a, B-2, and marginal zone (MZ) B cells in the spleen and B-1a, B-1b, and B-2 cells in the peritoneal cavity can be distinguished.

In adulthood, B cells continuously arise from hematopoietic stem cells (HSCs) that reside mainly in the bone marrow (BM). In contrast, in the fetus the liver is the major B cell generating organ (2). In earlier studies, it was suggested that the B-1a cell population could only develop during the fetal stage and is maintained by self-renewal (3). This was based on findings where B-1a cells could not be detected after adoptive transfer of adult BM cells. However, more recently several groups have demonstrated the existence of BM-residing precursor cells that could give rise to B-1a cells even in adult mice (4, 5). Thus, adult BM sustains the potential to give rise to B-1a cells. Nevertheless, a contribution of such precursor cells to the pool of adult B-1a cells in unmanipulated adult mice has not been clearly shown yet.

Development of B-2 cells in the adult BM has been studied very extensively. Cellular markers and status of immunoglobulin rearrangement at the various stages have been established and correlated. In brief: the early committed B cell precursors known as proB/preB-I cells can be identified by the expression of c-kit and B220 and partial lack of CD19 (6). This stage is followed by the stage of large cycling pre-B-II cell (B220^+^CD25^+^), then of the stage of small preB-II cell (B220^+^CD25^+^) and finally the stage of the immature B cell (B200^+^sIgM^+^). At such developmental stages, rearrangement and expression of the immunoglobulin gene segments takes place in an ordered fashion i.e., DJ and VDJ at the pro-B/preB-I, preB cell receptor expression at the large preB-II and rearrangement of gene segments of the light (L) chain loci at the small pre-B-II cell stage. Subsequently, immature B cells are the first B cells to express surface IgM. They emigrate from the BM and complete their development to mature B cells (CD19^+^CD93^−^IgM^+^) in the peripheral lymphoid organs in two clearly discernable transitional steps: T1, CD19^+^CD93^+^IgM^+^CD23^−^, and T2, CD19^+^CD93^+^IgM^+^CD23^+^ (7).

To study B cell development, we have established a new recombinant mouse system—B-Indu-Rag1 (8). In such mice, B cell development is arrested in the pro-B cell stage due to an inversion of the coding exon 2 of the recombination-activating gene 1 (*Rag1*). In addition, the inversion is flanked by two loxP sites in opposite direction. These mice also express the recombinase Cre fused to a mutant estrogen receptor. It is driven by the B cell-specific *mb1* promoter. Hence, B cell development can be induced specifically. Upon application of Tamoxifen (TAM), the coding exon of the *Rag1* gene is inverted and expression of Rag1 is activated. Thus, B cell development starts in a synchronized way. Since Rag1 expression is initiated in a precursor cell that is not self-renewing, only a single wave of B cell development can be induced. Using such mice, it is possible to monitor several parameters of B cell development, like the minimal timing that developing B cell require for completion of particular stages, as well as the time that the majority of developing B cells remain in a particular stage. Only a rough estimate exists for the time that such processes require. Data from fetal liver exist on the timing required for B cell development from the c-kit^+^ proB cell to the first IgM^+^ B cell. It was estimated of roughly 6–7 days (9, 10).

The locus encoding the V regions of the murine heavy (IgH) chain contains 15 different VH families comprising more than 100 different individual gene segments (11, 12). It was claimed that during fetal development of B cells, the V gene segments most proximal to the constant (C) region are used first (13). The explanation given suggested that activation of particular V gene families for rearrangement might require different signals. Proximal V gene segments should become accessible first because V genes more distal to Cμ would require the presence of IL-7 for accessibility (14). Similar suggestions were made for early progenitors during adulthood (15, 16). We wanted to confirm these findings in the B-Indu-Rag1 mice, since early after induction of B cell development in such mice, the rearrangement process should be in synchrony (8). Rare rearrangements during the early phase might be diluted out under steady-state conditions or might be lost due to selection processes. Here, they should become detectable shortly after application of the inducer TAM in such mice.

In the present work, we used the B-Indu-Rag1 model to estimate the length of time that a B cell precursor requires to pass the various stages of B cell development in the adult. We could further show that, indeed, at the earliest time points after induction, preferentially the V gene segments most proximal to Cμ are rearranged. Finally, by adoptive transfer experiments into sublethally irradiated recipient mice, we show that B-1 cells arising from adult progenitors are able to contribute to the B-1 cell pool of adult mice.

## Materials and methods

### Mice

B-Indu-Rag1 mice were described in Duber et al. (8). CB20, Igα^−/−^ (17) and RAG1^−/−^ (18) mice were obtained from the Basel Institute for Immunology and kept under specific pathogen free conditions (SPF) in the animal facility of the Helmholtz Centre of Infection Research. BALB/c mice were purchased from Janvier (Le Genest-Saint-Isle, France). Mice, aged between 8 and 14 weeks were used for the experiments. For induction of B cell development or as control, 400 μl of a 20 mg/ml solution of TAM (Ratiopharm) in ClinOleic (Baxter) was administered orally. All animal studies were performed in strict accordance with German Animal Welfare legislation. All protocols were approved by the Institutional Animal Welfare Officer (Tierschutzbeauftragter), and necessary licenses were obtained from the regional license granting body (LAVES, permission number 33.9-42502-04-11/0390).

### Flow cytometry and adoptive cell transfer

Single cells were obtained by flushing lymphoid organs or the peritoneum of mice with ice-cold IMDM (Iscove's modified Dulbecco's medium, GibcoBRL/Invitrogen). Erythrocytes were lysed by incubation with ACK buffer (0.15 M NH_4_Cl, 10 mM KHCO_3_, 0.1 mM Na_2_EDTA, pH 7.2) for 2–3 min on ice. For the isolation of cells originating from blood, cardiac-blood was collected into 500 μl of PBS including 50 μl 1:100 diluted heparin 25,000 (Ratiopharm). Erythrocytes were lysed by adding Erythrocyte-lysis buffer (2.06 g Tris-base, 7.49 g NH_5_Cl in a total volume of 1 l H_2_O, pH 7.2) for 5 min at RT. Cells were centrifuged for 5 min at 1,000 rpm 4°C. The supernatant was discarded and the steps were repeated until the cell pellet was white. For T cell transfer, 3 × 10^6^ splenocytes, isolated from B cell-free Igα^−/−^ mice (10–14 weeks old) were injected i.v. in a volume of 100–200 μl sterile PBS into CB20 or RAG1^−/−^ mice and were analyzed by flow cytometry to confirm the composition (40–50% of T cells). For flow cytometric analysis, cells were resuspended in 1x PBS containing 2% FCS and 0.5 mM EDTA. Cells were stained with the following antibodies conjugated with flourophors or biotin directed against: CD11b, CD3, CD4, CD5, CD8, CD25, CD40, CD23, CD93 (all eBioscience), B220, CD19, CD43, CD21, IgMa, IgMb (all BDPharmingen), IgD (clone 1.19, prepared in our lab). Biotinylated antibodies were revealed using various streptavidin conjugates (BD Biosciences or Southern Biotech). BrdU labling at day 9 after TAM application and staining were performed exactly according to manufacturer's protocol (FITC BrdU Flow Kit, BDPharmingen). Flow cytometric analysis always included gating on lymphocytes, exclusion of doublets as well as exclusion of dead cells by DAPI staining. Analysis was carried out using an LSRII or Fortessa (Becton Dickinson). Data were analyzed using DIVA software (Becton Dickinson) or FlowJo. Cell sorting was carried out on a FACSAria (Becton Dickinson).

### Ig concentration measurements and ELISPOT

IgA concentration from intestinal wash out was determined by ELISA according to standard protocol (8) using goat anti-mouse IgA (Sigma) as coating antibody and peroxidase coupled goat anti-mouse IgA (Sigma) as detection antibody. Immunoglobulin concentrations from sera were measured using a kit according to the manufacturer's protocol (Mouse Immunoglobulin Isotyping Panel 6plex FlowCytomix Multiplex, eBioscience). Flow cytometry was carried out using analyser LSRII (Becton Dickinson). ELISPOTs were performed according to standard protocols (8). ELISPOTs detecting IgA and IgG secreting cells were performed according to the same protocol, using anti-IgA (clone C10-3, BD Pharmingen) or goat anti-mouse IgG (Sigma) as capturing antibodies and biotinylated anti-IgA (clone 11-44-2, eBioscience) or biotinylated goat anti-mouse IgG (MABTECH) as secondary antibodies.

### BM transfer

For adoptive transfer, 8–12 weeks old B-Indu-Rag1 mice were used as donors. BM cells of both femurs were prepared and 3 × 10^6^ cells were injected i.v. into sublethally irradiated (5 Gy) 8-week-old Rag1^−/−^ or CB20 mice. B cell development in the recipients was induced by TAM application 6 weeks after irradiation and cell transfer and analyzed after 3 weeks.

### Nucleotide sequence analysis of V_H_ genes

Total RNA from BM cells from induced mice after 1–7 days and sorted BM cells from wild-type (Wt) mice was isolated using RNeasy MiniKit (Qiagen). cDNA synthesis was performed using RevertAid Reverse Transcriptase (Fermentas) using IgM-specific primers: 5′-ATGGCCACCAGATTCTTATCAGA-3′ and 5′-GAGGTGCAGCT GCAGGA GTCTGG-3′. For template library generation of IgM sequences, PCR with a primer binding in the constant cμ region (5′-CTATGCGCCTTGCCAGC CCGCTCAGA(MID)ATTTGGGAAGGACTGA-3′) in combination with a primer binding in the V_H_ region of all V_H_ genes 5′-CGTATCGCCTCCCTCGCGCCATCAGGA GGTGCAGCTGCAGGAGTCTGG-3′ was performed. MID (multiple identifier) consists of 4 nucleotides in 9 different variations for identification of single samples in a single lane. PCR conditions were as follows: 95°C, 1 min; 35x (95°C, 30 s, 55°C, 40 s, 72°C, 40 s); 72°C, 10 s. Amplicons were purified by gel extraction (QIAquick Gel Extraction kit; Qiagen). Sequencing by the 454 method and data analysis was done exactly as described (19).

### Curve fitting

To model the development of the frequency of B cell subsets over time, three parametric functions were considered: The constant function ***f***(*t*) = ***a*** with the only parameter *a*, representing no (significant) change over time the linear function ***f***(*t*) = ***a* · *t*** + ***b*** with two parameters for a linear increase or decrease over time and the bell-shaped parametric curve ***f***(*t*) = ***a* + *b*** · exp((***t* − *c*)^2^/*d*)**. Parameters **a** and c can shift the curve in y- or x-direction, respectively; parameters **b** and **d** can stretch the curve in y- or x-direction, respectively. The bell shaped curve models an increasing behavior followed by a decrease or vice versa. A least squares approach was used to fit the curves to the data. In the case of the bell-shaped function being nonlinear in its parameters, the fitting was carried out with the function optim within the statistics software R (20). It would not be meaningful to base the choice of the best of the three models on the error, i.e., the mean squared error because the most complex model—here the bell-shaped function—would always yield the smallest error. The corrected Akaike information criterion (AIC) (21) is a method used for choosing the most suitable model among—usually linear—parametric functions of different complexity in terms of the numbers of parameters. The corrected AIC evaluates the different parametric functions based on the variance of the residuals, the number of parameters and the sample size. Especially for small sample sizes as considered here, using the residuals directly would favor parametric functions with a tendency to overfitting. Therefore, the variance of the residuals was estimated based on leave-one-out cross-validation. With leave-one-out cross-validation the residual at each point for a parametric function is computed by removing the point from the data set, computing the corresponding parameters of the function based on the remaining data points and then taking the residual of the point that was not involved in the parameter estimation. This is repeated for each data point and each of the three parametric functions. The bell-shaped parametric curve is not only a nonlinear function but also nonlinear in its parameters, making it much more flexible than the constant and the linear model. To account for this extreme flexibility and not favor the bell-shaped curve, each of the four parameters of the bell-shaped function was counted twice for the corrected AIC.

### Statistical analysis

Statistical analysis was done using a *Mann-Whitney-U-test* or a two-tailed Student *t-test*. A *p* value < 0.05 was considered statistically significant.

## Results

### Development of B cell progenitors in the BM of adult mice

**Figure 1:**
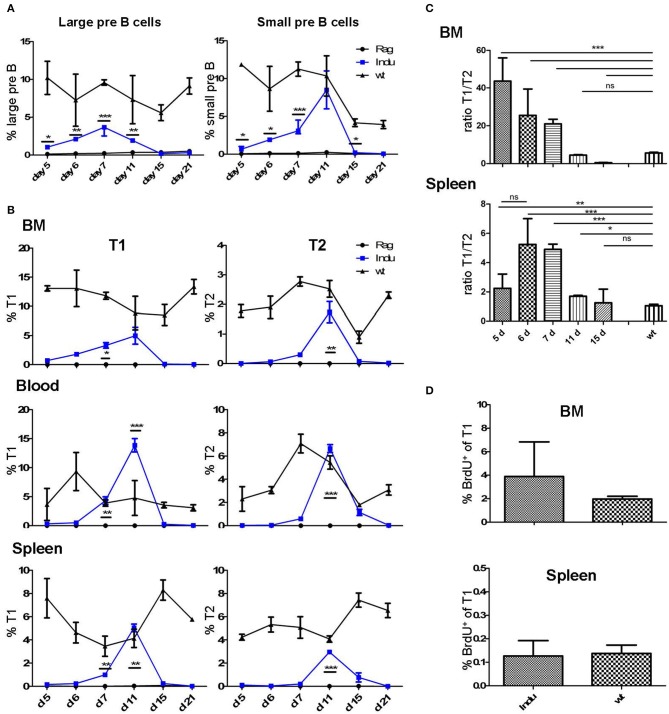
Progenitor and immature B cells after TAM-mediated induction of B cell development in different organs of adult B-Indu-Rag1 mice**. (A)** Large (left) and small (right) preB-II cells in BM at indicated time points after induction of B lymphopoiesis. **(B)** Kinetics of transitional B cell (T1 and T2) appearance at different anatomical sites (BM, peripheral blood, spleen) and time points post induction, as indicated. **(C)** Ratio of B cells with a T1 and T2 phenotype in BM and spleen of B-Indu-Rag1 mice at indicated time points after TAM administration. Note that the T1/T2 ratio of Wt mice is shown for comparison (right bar). **(D)** Cell proliferation of T1 cells from BM and spleen of TAM-treated B-Indu-Rag1 (left) and Wt (right) mice, as revealed by BrdU incorporation *in vivo*. Labeling was carried out exactly as described by the vendor. BrdU 2 mg was given i.p. 9 days after application of TAM and the incorporation was analyzed 2 h later by flow cytometric analysis. **(A–D)**: Graphs show percentages of indicated B cell populations as median ± SEM (*n* ≥ 3 mice); asterisks in graphs indicate the level of significance (see methods for details). The experiments were carried out at least twice.

We intended to use the power of the B-Indu-Rag1 mice to establish or to confirm parameters of B cell development in the adult mouse. First, we estimated the timing that proB/preB-I cells need for their developmental progression to the subsequent stages of the B cell lineage. B-Indu-Rag1 mice were induced once with TAM and at different time points cells were isolated from different tissues and analyzed by flow cytometry (Supplementary Figure S1). A first increase in percentage of progenitor cell could be detected from day 5 on for the large preB-II cells (B220^+^AA4.1^+^CD25^+^CD40^−^IgM^−^; Figure 1A and Supplementary Figure S2). Similarly, a first increase could be observed for small preB-II cells (B220^+^AA4.1^+^CD25^+^CD40^+^IgM^−^; Supplementary Figure S2A) at day 6 (Figure 1A and Supplementary Figure S2A). At days 7 and 11 the respective subset reached maximum (Figure 1A and Supplementary Figure S2A). By applying least square analysis, a time span of day 8 to day 13 could be defined, in which the majority of small preB-II cells were found. Since B cell development was induced only once (i.e., by a single-dose administration of TAM), the percentages of the preB cell subsets declined after reaching the peak. No further differentiation from proB/preB-I cell is expected to replenish the preB-II cell compartment.

Immature B cells (T1 and T2) migrate out of the BM via the blood stream into the spleen where they further mature (22). Both subsets are CD19^+^AA4.1^+^ (Supplementary Figure S1B). Due to expression of CD23 and IgM it is possible to distinguish these two populations (23). After TAM application, the first T1 cells could be detected at day 6 in the BM, while T2 cells required one day more (Figure 1B and Supplementary Figure S2B). In blood, such cells appeared roughly at the same time: in contrast, appearance in spleen was detected with a delay of one-day for both subsets. Highest percentage of transitional B cells was found between day 10 and 12 in all locations analyzed. The peak at day 11 for blood and day 12 for spleen (Figure 1B) was confirmed by least square analysis (Supplementary Figure S2B). In addition, in all particular locations specific cells were detected first in the T1 and then in the T2 compartment. This confirms the notion of a stepwise maturation of transitional B cells from T1 to T2 (23, 24).

The sequential maturation also became clear, when comparing the relative cellularity in the T1 and T2 compartments. Due to continuous generation and maturation in adult WT mice, the ratio between the two different transitional B cell subsets remained equal over time. Since B cell maturation in B-Indu-Rag1 mice starts with the time of induction, the ratio changes over time (Figure 1C). Interestingly, in BM the ratio declined nearly to zero at day 15. This verified that transitional B cells first develop in the BM and later on migrate into the periphery (Figure 1C).

Proliferation of T1 and T2 cells without exogenous B cell receptor (BCR) stimulation has been described before (25). Proliferation of such cells suggested expansion of both B cell populations during this stage. To confirm population expansion by proliferation of transitional B cells in the B-Indu-Rag1 model, we used BrdU incorporation. We expected that proliferative expansion would take place before the population reaches peak levels. Thus, we analyzed the cells at day 9 after induction. In blood, no proliferating transitional B cells were detected. In contrast, strong proliferation of T1 B cells could be found in BM and minor proliferation in spleen (2 and 0.1%, respectively; Figure 1D). No BrdU incorporation in T2 cells could be observed under these conditions (data not shown).

### First detection of mature B cells as early as 11 days post-induction

**Figure 2:**
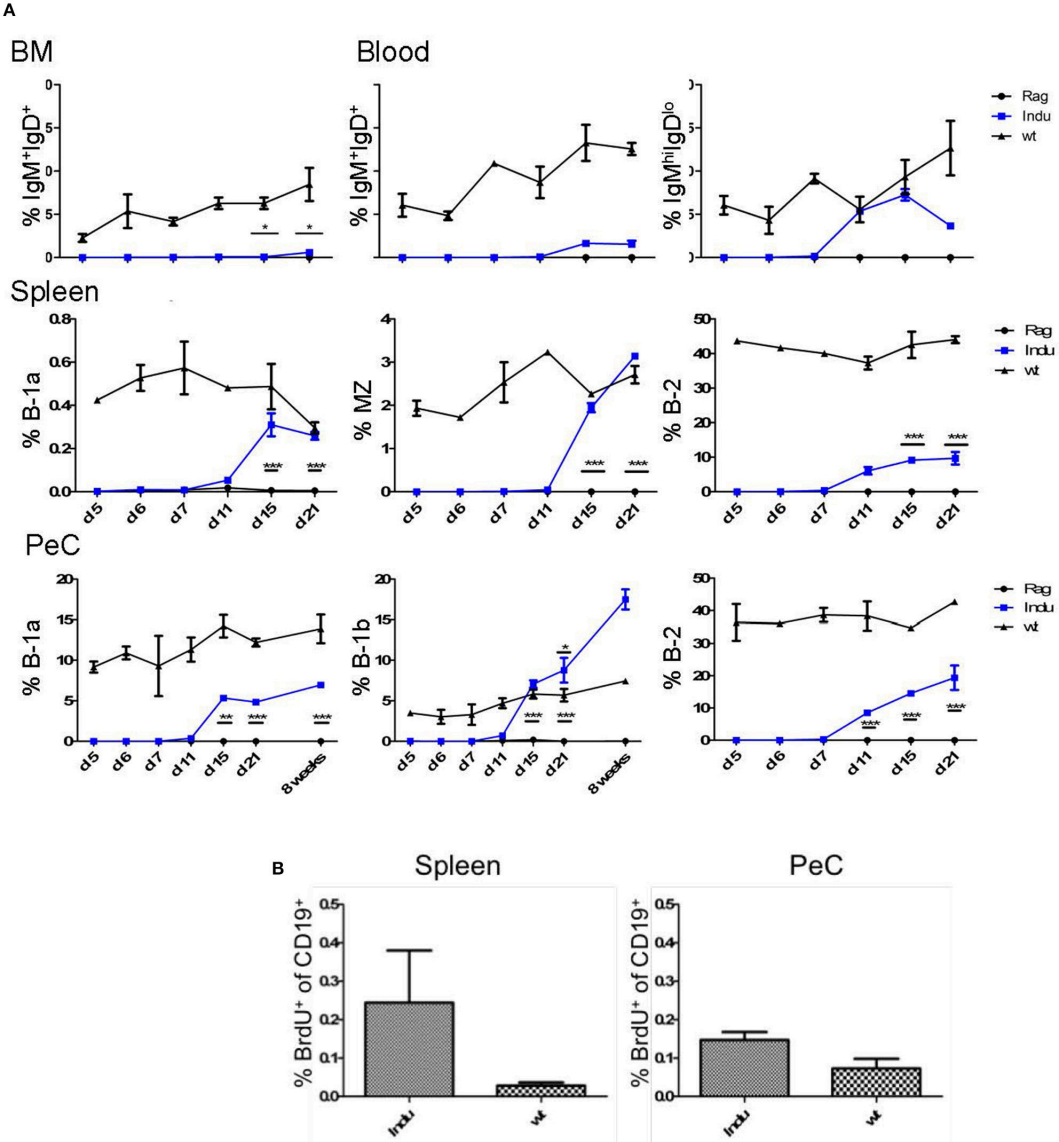
BM-derived mature B cells rise over time in B-Indu-Rag1 mice after TAM-mediated induction of B lymphopoiesis. **(A)** Graphs depict the kinetics of newly generated BCR^+^ B cell accumulation (IgM^+^IgD^+^, IgM^high^IgD^low^, B-1a, B-1b, B-2) at different anatomical sites [BM, blood, spleen, peritoneal cavity (PeC)], as indicated. **(B)** Cell proliferation of CD19^+^ B cells from spleen and peritoneal cavity of TAM-treated B-Indu-Rag1 (left) and Wt (right) mice, as revealed by BrdU incorporation *in vivo*. The proliferation of T1 was analyzed 9 days after induction. BrdU was given i.p. and 2 h later the incorporation was analyzed by flow cytometric analysis. **(A,B)** Mean percentages ± SEM (*n* ≥ 3 mice); asterisks in graphs indicate the level of significance (see methods for details). The experiments were carried out at least twice.

Immature B cells migrate from BM to the spleen to continue further maturation (22, 26). To determine the time frame for these events, the first appearance of mature B cells was analyzed. Mice were treated once with TAM and the presence of B cell populations in different locations was monitored in kinetics studies (day 5, 6, 7, 11, 15, and 21 after induction). In spleen, all mature B cells are CD19^+^, and due to their differential expression of CD21, CD23 and CD5, they can be divided into the different subsets (Supplementary Figure S3). All B cell subsets could be found in significant numbers at day 15 after TAM administration (Figure 2A and Supplementary Figure S4). While with time percentages of B-1a and MZ cells approached the percentages found in WT mice, the percentage of B-2 cells remained relatively low compared to WT mice. This might be due to the self-renewing capacity of B-1a and MZ B cells. By analyzing BrdU incorporation 15 days post induction in spleen and PeC, hardly any proliferating CD19^+^ B cells could be detected at these locations (Figure 2B). In general, the percentages of B-1a and MZ cells in spleen (induced mice: 0.3 and 3%, respectively) were much lower, as compared to the B-2 cell population (induced mice: up to 10%; Figure 2A; Supplementary Figure S4).

Some mature B cells, after maturation in the spleen, migrate back to the BM (24). Consequently, we analyzed the minimal time mature B cells require to develop and to migrate back to the BM. To this end, we divided B cells into two populations: both express CD19, but one population, mainly B-2 cells, are IgM^+^IgD^+^ and the other, consisting of B-1 cells, is IgM^hi^IgD^lo^. It is thought that especially IgM^+^IgD^+^ B cells in the BM are the major population that matures in the spleen and recirculates back to the BM (27). Indeed, we could show that this population increased over time (Figure 2A). The first mature IgM^+^IgD^+^ cells can be detected 15 days post induction in the BM (Figure 2A and Supplementary Figure S2). In general, and similar to WT mice, only very low percentages of IgM^hi^IgD^lo^ B cells could be detected in the BM (max. 0.6% in WT mice; data not shown). The major mature B cell population in the BM consists of IgM^+^IgD^+^ B cells. In induced mice, the IgM^+^IgD^+^ population rose only up to 0.6%, whereas it is roughly 7% in Wt mice (Figure 2A).

The main producers of natural IgM are B-1a cells. They can react to T cell-independent stimulation and act as innate-like cells (28). Their task in the immune system is to quickly produce antibodies upon infection. In contrast to B-2 cells, B-1a cells are self-renewing and their progenitors are mainly found in fetal liver. Nevertheless, also in adult BM some B-1a progenitors are present (5, 8, 29). Therefore, we expected to find newly developed B-1a cells in the periphery after induction (8). Similarly, we also expected to find B-2 and B-1b cells after induction. The latter is normally enriched in the peritoneal cavity. At this anatomical site, we could detect B-2 cells (CD19^+^CD43^−^CD11b^−^CD5^−^) first around 11 days after TAM induction, whereas the B-1 cell subsets were found roughly from day 15 onwards (Figure 2A and Supplementary Figures S4, S5). While the percentages of B-1a (CD19^+^CD43^+^CD11b^+^CD5^+^) and B-2 cells were not reaching the level of Wt mice, the percentage of B-1b cells (CD19^+^CD43^+^CD11b^+^CD5^−^) in the peritoneal cavity was rising above Wt-level as seen before (17% compared to 6% after 8 weeks, Figure 2A and Supplementary Figure S5) (8). Eight weeks after induction, also the absolute number of B-1b cells in induced mice was reaching the level of Wt mice. In contrast, the absolute numbers of the other subsets never reached WT-level.

Importantly, at this time point the increase of mature B cell populations cannot be attributed to cell proliferation, as we detected no increased BrdU incorporation (Figure 2B). Therefore, the question remained where the higher percentages, especially of B-1b cell population arise from over time. No progenitors can be detected after day 11 and the transitional B cell subsets also vanished at day 21 (Figure 2B).

### Ig's increase over time in serum and gut

**Figure 3:**
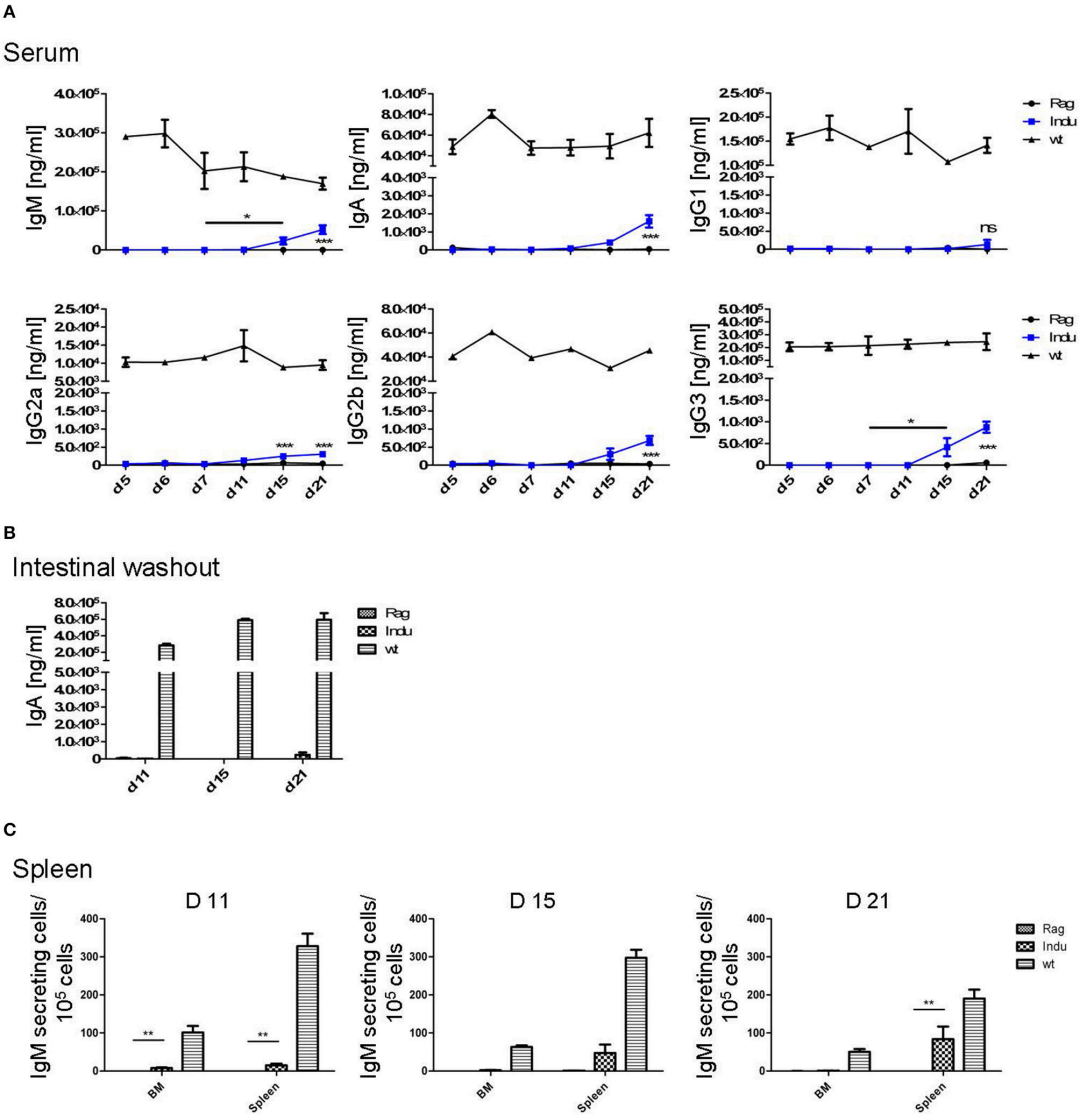
Induction of B lymphopoiesis in B-Indu-Rag1 mice correlates with increased Ig concentrations over time. Ig classes (IgM, IgA, IgG) and IgG subclasses (1, 2a, 2b, 3) in **(A)** serum and **(B)** intestinal washout of 3 mice per group were quantified by ELISA (ng/ml) at different time points after initiation of the experiment, as indicated. Blue squares: TAM-treated B-Indu-Rag1 mice; black circles: Rag1^−/−^; mice; black triangles: Wt mice. **(C)** Number of IgM-secreting cells among 10^5^ total cells from BM and spleen of TAM-treated B-Indu-Rag1 mice, as revealed by ELISPOT at indicated time points after initiation of the experiment. Data from Rag1^−/−^ and Wt mice are included for comparison (2–3 mice per group). **(A–C)**, mean values ± SEM; asterisks in graphs indicate the level of significance (see methods for details). The experiments were carried out at least twice.

To analyse the functionality of the B cell populations after TAM induction, we investigated their ability to produce Ig's. Thus, the Ig concentration in serum of the different mouse groups was determined. Similar to the increase of the B cell subsets in the different organs, Ig's became first detectable at day 15 after induction (Figure 3A). However, this was found only for IgM, IgG2a, and IgG3. The IgA and IgG2b subclasses could first be detected in significant amounts after 21 days of TAM induction. IgG1 was not increasing significantly at all. At the same time, the highest concentration could be detected for IgM with a tendency to reach WT levels. This is the only Ig that rised to a concentration of 50 μg/ml. All other Ig subclasses did not rise above 1 μg/ml within the observation time (Figure 3A). Obviously, efficient switching of B cells to other Ig subclasses was not supported in B-Indu-Rag1 mice, most likely due to the lack of T cell help.

Intestinal IgA might be mainly derived from B2 cells that were triggered in the Peyers Patches or mesenteric lymph nodes and, after differentiation, migrated into the lamina propria (LP). In addition, B-1 cells are claimed to be able to migrate to intestinal LP (24). For some time, it was believed that B-1 might be key players in intestinal immune responses by switching to IgA-secreting cells in a T cell-independent manner (24). However, recently it was shown that LP-derived IgA^+^ cells might not be derived from peritoneal B-1a, but rather from B-1b cells (30). To analyse the functionality of B cells in the induced mice in more detail and to investigate the kinetics of IgA accumulation in the gut, intestinal washout was analyzed at 11, 15, and 21 days after induction. In such mice, nearly no IgA in the intestinal wash out became detectable (Figure 3B). Only 230 ng/ml at day 21 after induction could be determined, as compared to 500 μg/ml in Wt mice during steady state.

IgM in serum of the induced mice was most likely due to an overall increase in the numbers of B cells actively secreting IgM. In contrast, a few B cells could be present that secrete high amounts of IgM, which then accumulate over time. Therefore, we performed ELISPOTs of B cells at different time points from spleen and BM, in the absence of any deliberate stimulation *in vitro*. The number of IgM secreting cells was increasing over time in the spleen (Figure 3C). While only 15 IgM-secreting cells/10^5^ cells were detectable in spleen 11 days after TAM induction, this number increased to 125 IgM-secreting cells/10^5^ cells at day 21. In the BM, at day 11, only very low numbers of IgM-secreting cells could be detected in the induced mice. This suggested that IgM-secreting cells in spleen might be short-lived plasma cells. Long lived plasma cells are expected to migrate to BM (Figure 3C). However, plasma blasts derived from B-1a cells might behave differently.

### T cells help to increase B cell numbers mainly in spleen

**Figure 4:**
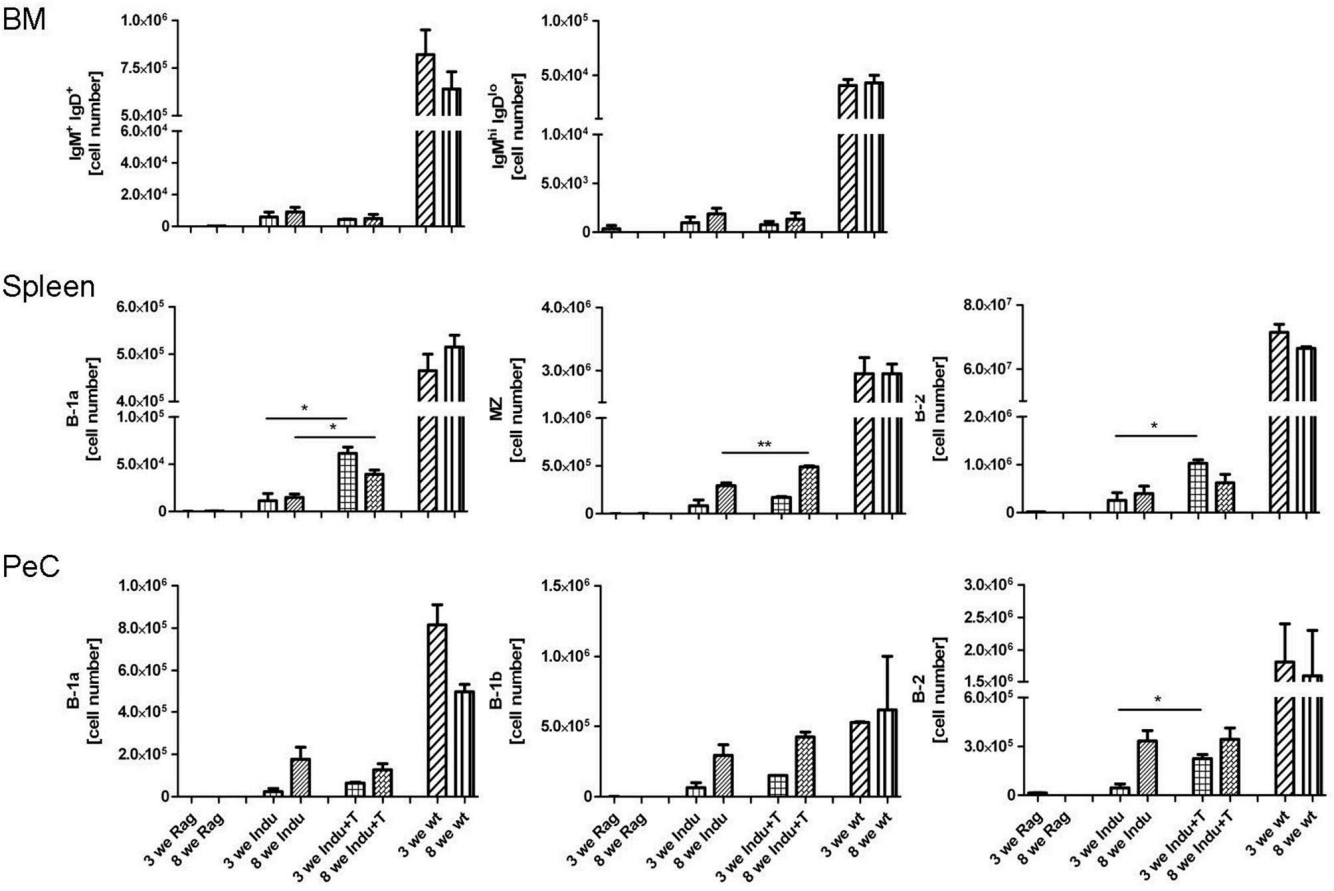
Adoptive T cell transfer increases B cell numbers in spleen of TAM-treated B-Indu-Rag1 mice. As indicated, shown are absolute numbers of different BCR^+^ B cell populations (IgM^+^IgD^+^, IgM^high^IgD^low^, B-1a, B-1b, B-2) at different anatomical sites (top: BM; middle: spleen; bottom: peritoneal cavity, PeC) and 3 and 8 weeks after initiation of the experiment. Note that, in addition to cohorts of B-Indu-Rag1 mice ± adoptive T cell transfer 1 day prior to TAM administration (Indu; Indu+T), we included Rag1^−/−^ and Wt mice for comparison (*n* = 3–4 mice per group). Graphs depict mean values ± SEM; asterisks in graphs indicate the level of significance comparing “Indu” and “Indu+T”. The experiments were carried out at least twice.

To differentiate into an antibody secreting plasma cell or a memory B cell, B-2 cells require help from T cells. This takes place in secondary lymphoid organs, like spleen or lymph nodes. In addition, the cytokine-milieu that is available during the activation of a B cell is decisive for the class and subclass of Ig expressed after differentiation (31, 32). To analyse the T cell requirement for efficient development of such B cells, T cells were adoptively transferred into B-Indu-Rag1 mice 1 day before TAM administration. The presence of various B cell subsets was analyzed by flow cytometry 3 and 8 weeks after induction. In BM, nearly no mature B cells could be detected, independent of whether T cells were transferred or not (Figure 4). In spleen, B-1a cells were found in significantly higher numbers, when T cells were available (Indu+T) than without T cell transfer (Indu; Figure 4). For MZ B cells, this was only true for the later (8 weeks) time point, while B-2 cells were found to be increased at both time points (3 and 8 weeks). In peritoneal cavity, only a slight influence of the T cells on the numbers of B cells was observed (Figure 4).

**Figure 5:**
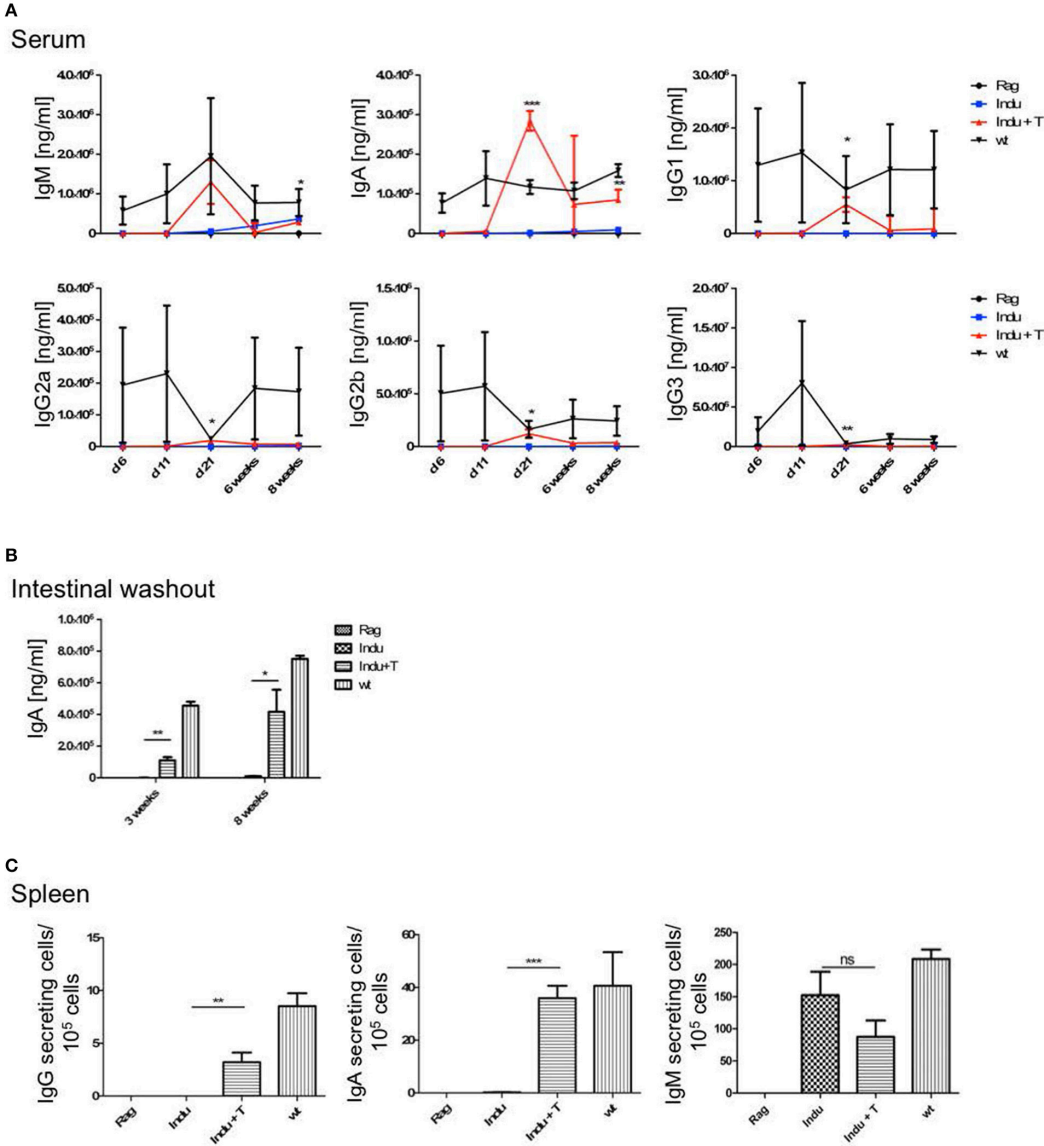
T cell transfer enhances Ig secretion by newly generated B cells. See legends to Figures 3, 4 for experimental details. In brief, at indicated time points after initiation of the experiment, mouse cohorts described in Figure 4 were subjected to ELISA-based Ig quantification in **(A)** serum **(***n* = 2–4 mice per group**)** and **(B)** intestinal washout **(***n* = 3). **(C)** Number of Ig-secreting B cells determined by ELISpot. Graphs depict Ig concentrations (ng/ml) or number of Ig-producing cells as median values ± SEM; asterisks in graphs indicate the level of significance comparing “Indu” and “Indu+T”.

As T cells obviously influence the development and differentiation of B cells, their effect on Ig secretion was investigated. Serum from mice taken at different time points after induction was analyzed. No differences in IgM concentrations between Indu and Indu+T could be observed (Figure 5A). In contrast, for all other Ig's (IgA, IgG1, IgG2a, IgG2b, and IgG3), marked differences were found. For each class or subclass, the concentration was significantly higher at day 21 post induction, when T cells were available. Analyzing later time points, no significant differences could be observed any more, except for IgA. Here, a significant higher concentration in T cell-reconstituted mice became apparent at 8 weeks post induction (Figure 5A).

Similarly, in intestinal wash outs, the IgA concentration was altered: at 3 or 8 weeks after TAM induction, mice that received adoptively transferred T cells consistently showed higher IgA concentrations, as compared to mice without T cells (Figure 5B).

In addition to higher serum concentration, the number of Ig-secreting cells was increased when T cells were available (Figure 5C). This held true for IgA and IgG, while numbers of IgM-secreting cells appeared largely similar, irrespective of whether or not TAM-induced mice were adoptively transferred with T cells (Figure 5C).

### B lymphopoiesis in adult BM can contribute to the peripheral B-1a cell pool

B-1a cells can arise from adult BM (5, 29), as was also shown using the B-Indu-Rag1 model (8). Similarly, in the present study we could show that readily detectable numbers of B-1a cells can arise from adult murine BM, in which only B cell development is possible and T cells are completely absent. Such adult BM-derived B-1a cells were functional, as they spontaneously secrete IgM, show PtC specificity and can survive for extended time periods in Rag1^−/−^ recipients after adoptive transfer (8). Thus far, the question whether B-1a cells still arise in adult mice was mainly approached in lymphopenic mice. Whether they also arise in a normal immunoproficient environment, is still not completely answered. In an effort to experimentally recapitulate physiological conditions as closely as possible, Rag1^−/−^ or CB20 mice (IgM^b^) were sublethally irradiated and subsequently reconstituted with total BM cells isolated from B-Indu-Rag1 mice (IgM^a^). Mice were kept for 6 weeks to allow recovery and re-establishment of a functional BM environment. Then, mice were induced once with TAM, followed by flow cytometric analysis of B cell subsets in peritoneal cavity after 21 days.

**Figure 6:**
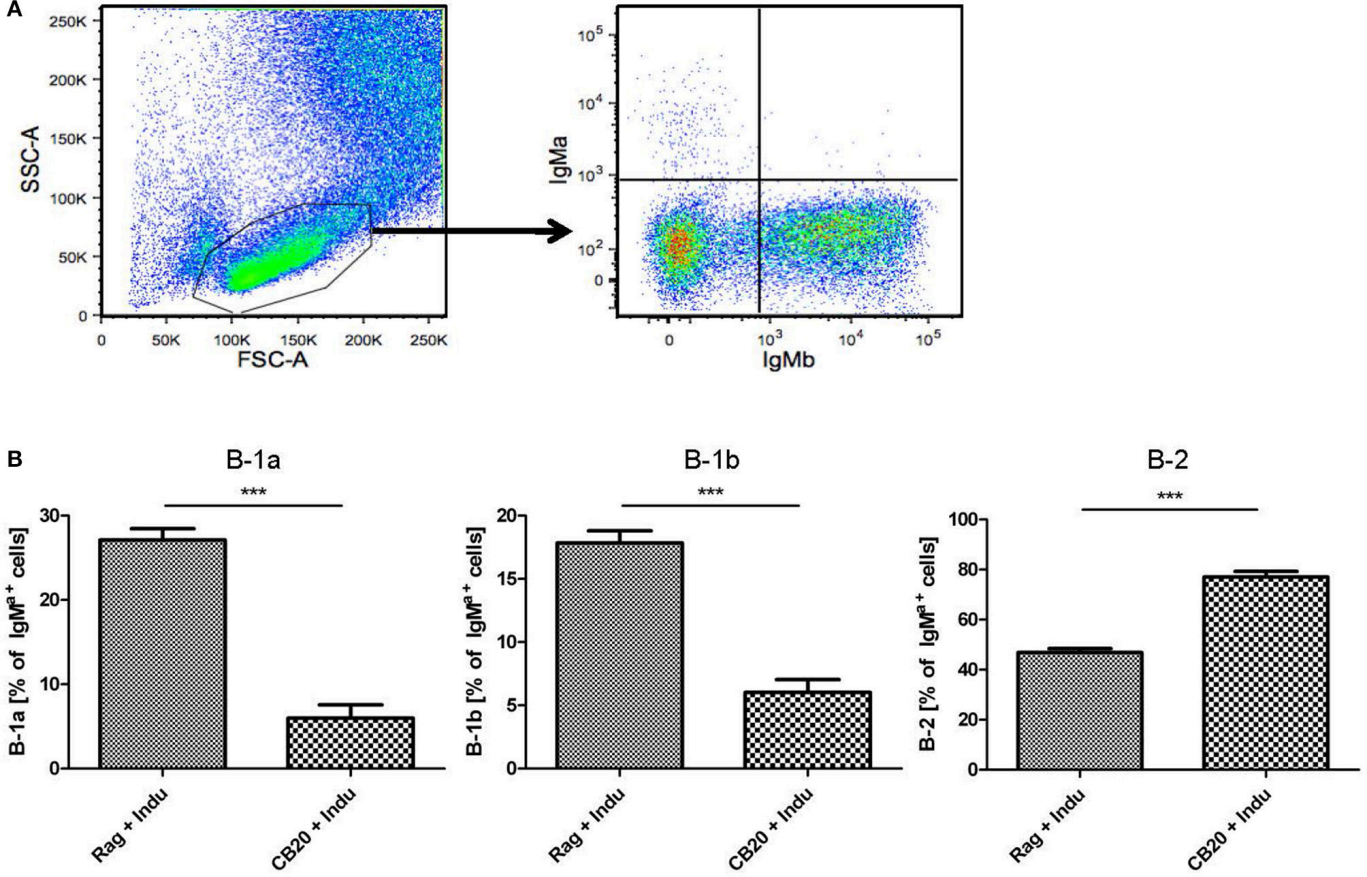
B lymphopoiesis in the adult BM can contribute to the mature B-1a cell pool. Tracking newly generated B-1a cells derived from adult BM. After irradiation conditioning, CB20 mice (IgM^b^) or Rag1^−/−^ mice (no endogenous IgM^+^ cells) were reconstituted with B-Indu-Rag1-derived BM (IgM^a^). 6 weeks later, cohorts of chimeric CB20 recipients were administered with TAM to induce B lymphopoiesis from engrafted B-Indu-Rag1 BM. **(A)** Dot plots depict representative flow cytometry of IgM^a^ and IgM^b^ surface expression among gated viable cells (FSC/SSC) among peritoneal exudate cells from CB20 mice (IgM^b^) at day 21 after TAM treatment. **(B)** Tracking mature IgM^a+^ B cell subsets (B-1a, B-1b, B-2) among peritoneal exudate cells of chimeric Rag1^−/−^ (left) and CB20 (right) mice. Graphs depict the percentage of B-Indu-Rag1 BM-derived IgM^a+^ cells as median values ± SEM; asterisks in graphs indicate the level of significance, as indicated. (*n* = 5 mice per experimental group).

As expected, the majority of B cells in the peritoneal cavity of CB20 recipients was derived from endogenous IgM^b^ progenitors (Figure 6A). However, we could also detect IgM^a+^ B cells derived from the donor BM (Figure 6A). In immune-compromised Rag1^−/−^ mice, in which the endogenous BM does not contribute to B cell development, B-1 cells dominated over B-2 cells (Figure 6B). Similar results had been obtained in TAM-treated B-Indu-Rag1 mice without T cell transfer (Figure 2). In contrast, in CB20 mice with a functional endogenous B cell compartment, nearly all newly developed donor-derived B cells could be assigned to the B-2 cell subset (77%). Nevertheless, donor-derived B-1a cells as well as B-1b cells could be found in the peritoneal cavity of such mice. The percentage of B-1a and B1b cells was much lower, when B cell development was induced in mice with endogenous B cell development (B-1a: 27 to 6%; B-1b: 18 to 5%, Figure 6B). A similar situation was observed in the spleen: nearly all B cells were IgM^b+^ as they arose from recipient progenitors and only a minor population was donor-derived (data not shown). Taken together, precursors in the adult BM most likely continued to contribute to the mature peripheral pool of B-1a cells. Thus, our data indicated that the development of B-1 cells is supported in the adult, even when a complete immune environment exists. However, and as expected, B-1 cell development was severely downscaled under these conditions (Figure 6B), perhaps due to competition with developing B-2 cells.

### Preferential usage of proximal V_H_ genes in the adult BM

**Figure 7:**
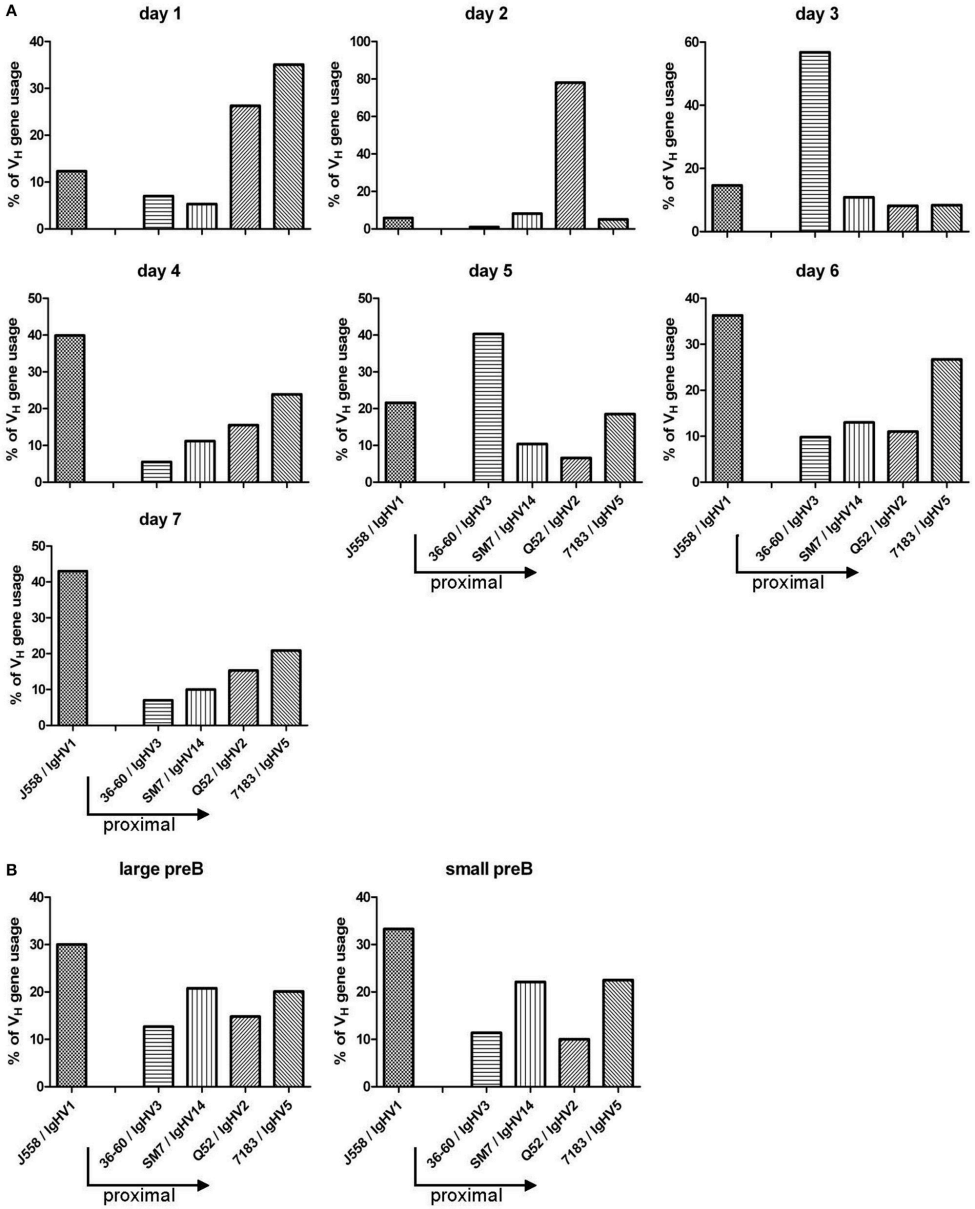
TAM-induced early B cell development in the adult BM is strongly biased toward VDJ rearrangements that involve proximal V_H_ gene segments. **(A)** Kinetics of V_H_ gene usage in the BM of B-Indu-Rag1 mice after induction of B lymphopoiesis, as revealed by analysis of sequenced RT-PCR products at indicated time points after TAM administration. Mean number of analyzed sequences: 407. **(B)** Quantification of V_H_ gene usage in FACS-purified populations of large and small preB-II cells from BM of Wt mice (BALB/c) with continuous B cell development. Mean number of analyzed sequences: 472. Arrows in graphs indicate the position of individual V_H_ gene families relative to the D/J clusters of the murine IgH gene locus (proximal to D/J: 36-60, SM7, Q52, 7183; distal: J558).

In B-Indu-Rag1 mice, shortly after TAM-mediated induction of B cell development, the process of Igh chain rearrangement to generate functional pre-BCRs should be in synchrony (8). Such initial VDJ recombination processes might be obscured under steady-state conditions in WT mice. Therefore, we next analyzed the rearrangement kinetics during TAM-induced B lymphopoiesis in the BM of B-Indu-Rag1 mice. Total BM cells were harvested daily from day 1 after TAM administration up to day 7. Gene-specific RT-PCR to assess V_H_ gene expression after rearrangement was carried out. For analysis, sequences of the PCR products were established. In addition, sorted large and small preB cells (Supplementary Figure S2A), isolated from control WT (BALB/c) BM cells were included for comparison. Interestingly, the experiment revealed that the preference of V_H_ usage varies over time (Figure 7A). At early time points, relatively few rearrangements involved the IgH_V1_ (former J558) family. This is the largest family that is found 5′ and most distal from the D and J gene cluster. In contrast, the proximal V_H_ families were found to be predominantly rearranged. At later time points, however, almost half of the detected VDJ rearrangements involved the IgH_V1_ family. Similar data had been described for B cells derived from fetal liver (13). When large and small preB-2 cells subsets from WT mice were analyzed for comparison (i.e., developing B cells in steady state), the data were comparable to those obtained at late time points from BM cells of TAM-induced B-Indu-Rag1 mice (Figure 7B). Thus, our experiments using the B-Indu-Rag1 model confirmed the notion that B cells at their initial developmental phase preferentially engage proximal V_H_ gene segments for rearrangement. Only when rearrangement is not taking place at this initial stage they also deviate usage to the distal V_H_ families.

## Discussion

The Indu-Rag1 mouse represents a highly versatile system to study various aspects of T and B cell development. For instance, when the Indu-Rag1 mouse is crossed to a mouse expressing a fusion of the Cre recombinase with a mutated estradiol receptor driven by the ubiquitously active Rosa26 promoter, induction of Cre activates Rag1 expression in HSCs. Since such cells are self-renewing, continuous T and B lymphopoiesis can be initiated. This approach has been recently used to characterize IL-17-producing γδ T cells (33) and early developmental stages of thymus-derived FoxP3^+^ T cells (34). In the present work, the Cre recombinase is driven by the B cell-specific mb-1 promoter. Cre is flanked on both sites with a mutated estradiol receptor, which renders its expression tightly controlled and inducible by TAM. By and large, no B cell development can be observed under non-inducing conditions, consistent with the severe developmental block at the pro/preB cell stage. TAM application leads to a single wave of development because pro-B cell precursors with activated *Rag1* gene expression are not self-renewing. Thus, they will be quickly exhausted. This B-Indu-Rag1 model has previously been successfully used to further corroborate the hypothesis that functional B-1a cells can develop from progenitors residing in the adult BM (8).

These previous experiments on B cell development in B-Indu-Rag1 mice involved several rounds of TAM administration (8). In contrast, in the present work, TAM was administered as a single dose, resulting in a single wave of B cell development. This allowed us to show that pro/preB cells differentiate into large and small preB-2 cell subsets within 5 and 6 days, respectively. The peak of these two BM subsets was detected 7 and 11 days after TAM induction, respectively. A clear order of differentiation could be observed. First, the large preB cells develop and later on the small preB cells. Small preB-II cells are known to differentiate from the proliferating large preB cell population. Nevertheless, differentiation from large to small preB-II cells appears to occur almost at the same time. Apparently, some of the large preB-II cells had quickly and functionally rearranged their IgH chain locus. This would predict that such large preB-II cells use mainly the proximal V_H_ gene segments. This hypothesis could be tested in future studies by establishing the V_H_ usage of isolated cell subsets. Additionally, some cells might be able to quickly proceed to generate a functional IgL chain. Again, one would predict that mainly the IgLκ locus is rearranged under these conditions (35).

A similar order of first appearance could be observed for transitional B cells: BM, T1: day 6, T2: day 7; spleen, T1: day 7, T2: day 8. Both T1 and T2 subsets peak at day 11 or 12 post induction. However, in contrast to the T1 subset, T2 cells remain detectable up to day 15 in blood and spleen. The fact that T1 cells disappear first is consistent with the notion of stepwise maturation of transitional B cells and their precursor-product relationship (23, 24). Clearly, only one wave of development takes place, as beyond 21 days post induction, no progenitor subsets can be detected anymore. This is also reflected by the T1/T2 ratio. By that time all progenitors have differentiated into mature B cells.

The first immature IgM^+^ B cells (T1) can be detected 6–7 days post induction. This is consistent with previous data obtained for B cell development in fetal liver. Here, the first c-kit^+^ progenitors can be detected at day 11 after fertilization (9). Six days later, the first IgM^+^ B cells can be detected in the fetus (10). This kinetics is very similar to the data obtained in the present work: Cells expressing c-kit^+^ can be found at day 0 of induction and the first IgM^+^ immature B cells became detectable 6 days later.

After TAM administration, the mature B cell compartment in peripheral organs increased starting with day 11, indicating that mature B cells (especially B-2 cells) need a minimum of 11 days for full maturation.

We could clearly show that the observed increase in cell appearance is not due to cell proliferation, as neither transitional B cells nor mature B cells show higher proliferation, as compared to fully immunocompetent WT mice in steady state. For transitional B cells, it was shown before that the T2 subset proliferates to a higher extend, when the BCR is stimulated (25). This was not the case in the present study, as no proliferation of T2 cells could be observed (data not shown).

Interestingly, B-1 and MZ B cells appear later than B-2 cells. This could indicate that the differentiation of such B cells requires more time. However, there is no indication for such scenario. More likely is that the precursor frequency for such cells is lower than for B-2 cells. Therefore, it requires more time for these cells to accumulate in significant numbers for detection.

Eight weeks after induction, B-1a and B-1b cells can still be found in elevated percentages in peritoneal cavity. In contrast, the B-2 cell population was found to be reduced. This is consistent with the notion that B-1 cells are self-renewing, whereas homeostasis of the B-2 compartment critically depends on the continuous replenishment from precursor cells.

The higher percentage of B-1b cells accumulating in TAM-induced mice compared to WT mice is consistent with data shown before (8). None of the other B cell populations in the peritoneal cavity increased to the same extend. The reason for the selective increase in the B-1b subset is still unclear. A possible explanation could be that the recently described B-1 precursor in BM leads mainly to B-1b cells (5).

By ELISPOT assays the first IgM secreting cells could be detected by day 11. Apparently, this does not lead to a significant increase in secretory IgM. At present, it is unclear whether these IgM-producing cells are of B-1a or B-2 origin. Under the given experimental conditions, both B cell subsets are likely to have the potential to give rise to short-lived plasma cells (36). No IgM-secreting cells could be found in BM at that time point.

Despite the presence of serum IgG and IgA, no Ig's could be detected in the intestinal wash out. This finding appears surprising, considering that the gut-associated lymphoid tissue is well-known to support T-independent Ig class switch recombination (37, 38). It might well be that, at the time point selected for our analysis, IgA-secreting cells have not colonized the intestinal LP yet.

A recent publication employing humanized mice indicated that human T cells are strictly required for the terminal differentiation of mature B cells. The absence of T cells resulted in a developmental arrest at the transitional stage (39). Based on the present work, it appears that the findings for human cells in mice does not extend to the development of murine B cells in their natural environment. Indeed, our experiments in TAM-treated B-Indu-Rag1 mice provided no evidence for a developmental block at the transitional B cell stage.

Interestingly, a T cell-effect on splenic B-1a cells could be observed. The B-1a numbers were significantly increased in B-Indu-Rag1 mice that received adoptively transferred T cells prior to TAM administration, as compared to TAM-treated mice without T cell transfer. In contrast, the population size of peritoneal B-1a cells appears to be completely independent of T cells. The reason underlying this T cell-effect in the spleen is at present unclear. It is known that B-1a cells react to their specific antigen in a T-independent manner (40). In addition, the differences between B-1a cell populations from spleen and peritoneal cavity are still ill-defined. Possibly, migration of B1a cells to the spleen is supported by T cells, a scenario that we have observed before (Roy, unpublished).

It is known that the cytokine milieu, which critically determines the outcome of Ig class switch recombination in activated B cells (31, 32) is at least in part dependent on T cells. Thus, the appearance of some of the Ig classes is highly dependent on the presence of T cells (41). Therefore, the serum Ig concentrations of TAM-induced mice that received adoptively transferred T cells, were additionally compared to sera of TAM-induced mice without T cell transfer. After 21 days, at which time point the first significant amounts of Ig's could be detected after TAM-mediated induction of B cell development, the Ig serum concentration was higher, when T cells were present. This correlated with increased numbers of Ig-secreting B cells in such mice. Even for intestinal wash out, it could be shown that the IgA secretion is highly dependent on T cells (37). Thus, still a high number of B cells do require T cells for the switching to IgA-secretion, and possibly also for migration and homing to the gut.

Nevertheless, while these experiments underscore the requirement for T cell help in Ig secretion, it appears that only short-lived plasma cells develop under these circumstances. Except for IgA, the increase in serum Ig was transient and lost at later time points of our kinetics analysis. Continuously high IgA concentration in the presence of T cells could potentially be attributed to the continuous immune stimulation provided by commensal bacteria located in the gut of SPF mice. These stimuli are supposed to activate B cells in a T cell-independent manner (38). This is obviously not sufficient for an increase in IgA, neither in the intestinal washout, nor in serum. The requirement of T cells for B cell maturation was again be confirmed by ELISPOT.

Under our experimental conditions, most Ig levels never reach normal levels. This is in contrast to mice in which B cell development had been stopped or was shown to be defective after birth. Such mice show a large portion of activated B cells and serum levels and plasma cell numbers are normal or even enhanced (42, 43). Why the B cell compartment is not filled up after induction and does not behave as in such lymphopenic mice, is unclear. A longer observation period might be required. Alternatively, the receptor repertoire under our conditions might be to restricted for a efficient expansion although previously we could show that the expected specificities are found in B-Indu-Rag1 mice (8).

The present study and previous publications (5, 8, 29, 44) indicate that B-1a cells can develop also from adult BM. As these B-1a cells are developing in an immunodeficient environment, it could be still argued that the adult BM might not give rise to these B-1a cells in a complete and functional immune environment. Therefore, we established BM chimeras in sublethally irradiated mice. We argued that such mice recover normal homeostasis within 6 weeks. Thus, Tam-induced B cell development occurs in a “quasi-normal” environment. Indeed, B-1a cells were observed that were derived from newly induced B cell progenitors under these conditions. However, when other competing B cells are available, it appears that such progenitors are more likely to develop into B-2 than into B-1 cells. This confirms data obtained by other groups on the presence of N-nucleotide additions of V-D-J junctions of B cell receptors from B1a cells of aged mice. This was taken as evidence for a contribution of newly formed adult B cells to the B-1a pool (44, 45). The exact mechanisms that govern the B-2/B-1a lineage fate decision in the adult BM under fully physiological conditions remains to be determined. Recently, Lin28b has been suggested to represent a B-1a cell-supporting transcription factor that drives such lineage decisions in fetal vs. adult mice (46, 47). In fact, some previous transfer experiments failed to provide evidence for the development of B-1a cells from the BM of adult mice (48), perhaps owing to the fact that the population size of newly developed B-1a cells in the adult is exceedingly small. This could also explain why in more recent experiments, no newly formed B1a cells derived from unmanipulated adult HSCs could be observed (49). In any case, in the present work, it could be clearly shown that also in immunocompetent mice B-1a cells do develop from adult BM-residing progenitors and that such newly generated cells contribute to the adult B-1a cell pool.

Previous studies showed that, in developing B cells in the young adult BM, the proximal V_H_ genes are used more often (15). This could be confirmed by using the B-Indu-Rag1 mouse. In the first days of B cell development after TAM induction, the proximal genes, close to the D and J clusters were preferentially found to be rearranged, whereas at later days the distal (J558; VH1) gene segments predominated. A clear shift from proximal to distal gene usage can be seen in our kinetics studies, as there are by day 7 post induction around 40% of gene segments used from the V_H1_ (J558) family, whereas it is only 10% by day 1–3. The normal sorted large and small preB cells that were included as controls gave the expected results. We therefore feel that we can exclude a bias due to the use of RNA and RT-PCR for analysis. Although higher numbers of sequences might provide a clearer picture, it appears that an individual B cell precursor has the chance to rearrange a proximal V_H_ gene segment first. As the process is slow, only some cells will be able to successfully assemble such V_H_ genes and express a functional heavy chain. Such cells will have a head start for further differentiation. They are most likely identical with the pre-BI cells that reach the next differentiation stage within the shortest time observed in the present work. Cells that require more time for IgH chain rearrangement have then the complete repertoire available, since the locus is now completely open due to cytokine activity or other not yet defined signals. As in normal WT mice B cell development is not synchronized, this could not be clearly defined in earlier experiments. Along this line, it was also shown before that in B cell precursors isolated from Rag2^−/−^ mice the histone assembly of distal VH genes allowing their active recombination was highly dependent on IL-7 (14). In the present mouse model, it would be possible to investigate this phenomenon in more molecular details *in vivo*.

## Author contributions

A-MB, SD, BR, and IT designed, performed and analyzed the experiments. FK analyzed experiments and performed statistical analyses. AG, OP, KK, and SW conceived the research. SW guided its design, analysis, and interpretation. A-MB, AG, KK, and SW wrote the manuscript.

### Conflict of interest statement

The authors declare that the research was conducted in the absence of any commercial or financial relationships that could be construed as a potential conflict of interest.

## Supplementary material

Supplementary Figure S1 Flow cytometric gating for large and small preB cells as well as for T1 and T2 transitional cells. **(A)** Shown is an example of staining of BM cells from a B-Indu-Rag1 mouse analyzed 6 days after induction. Cells were gated for lymphocytes using forward and sideward scatter. Doublets were excluded by applying FSC area and heights against each other. Dead cells were excluded by gating for DAPI-negative cells. Those cells were then gated for B220^+^AA4.1^+^ cells for immature B cell population as shown and finally for CD25 and CD40. IgM bearing cells were excluded. **(B)** Shown is a representative staining for BM cells of an induced Indu-BRag1 mouse. Gating was done as in A. Transitional B cells amongst B cell progenitors could be differentiated into T1 and T2 as CD23^−^IgM^+^ (T1) and CD23^+^IgM^+^ (T2). Similar gating was performed for all tissues analyzed.

Supplementary Figure S2 Results of least squares fitting. In each case, a constant, a linear and a bell-shaped model were fitted. The lines shown correspond to the best model according to Akaike's information criterion. **(A)**. Frequencies of indicated B cell developmental stages based on data from Figure 1A were used and fitted by least square statistics. **(B)**. Data from Figure 1B were used and fitted by least square statistics.

Supplementary Figure S3 Gating strategy for mature B cells. Shown is a representative staining for cells isolated from spleen of an induced B-Indu-Rag1 mouse. Cells were gated for lymphocytes first using forward and sideward scatter. Doublets were excluded by applying FSC area and heights against each other. Dead cells were excluded by gating for DAPI-negative cells. Those cells were then gated for CD19 and furthermore classified according to the figure and CD23, CD21, CD5 expression.

Supplementary Figure S4 Gating strategy for peritoneal mature B cell populations. Shown is a representative staining for cells isolated from peritoneal cavity of an induced B-Indu-Rag1 mouse. Cells were gated for lymphocytes first using forward and sideward scatter. Doublets were excluded by applying FSC area and heights against each other. Dead cells were excluded by gating for DAPI-negative cells. Cells were further subdivided based on their expression of CD19, CD43, Mac-1 (CD11b), and CD5.

Supplementary Figure S5 Results of least squares fitting for mature B cells. Analysis was carried out like for Figure S2. Frequencies of indicated BCR^+^ B cell subsets based on data from Figure 2 were used and fitted by least square statistics.
