# Supplementary figures and images for: Induced B Cell Development in Adult Mice
Source: Front Immunol. 2018 Oct 31;9:2483. doi: 10.3389/fimmu.2018.02483 (PMC6220648; doi:10.3389/fimmu.2018.02483)

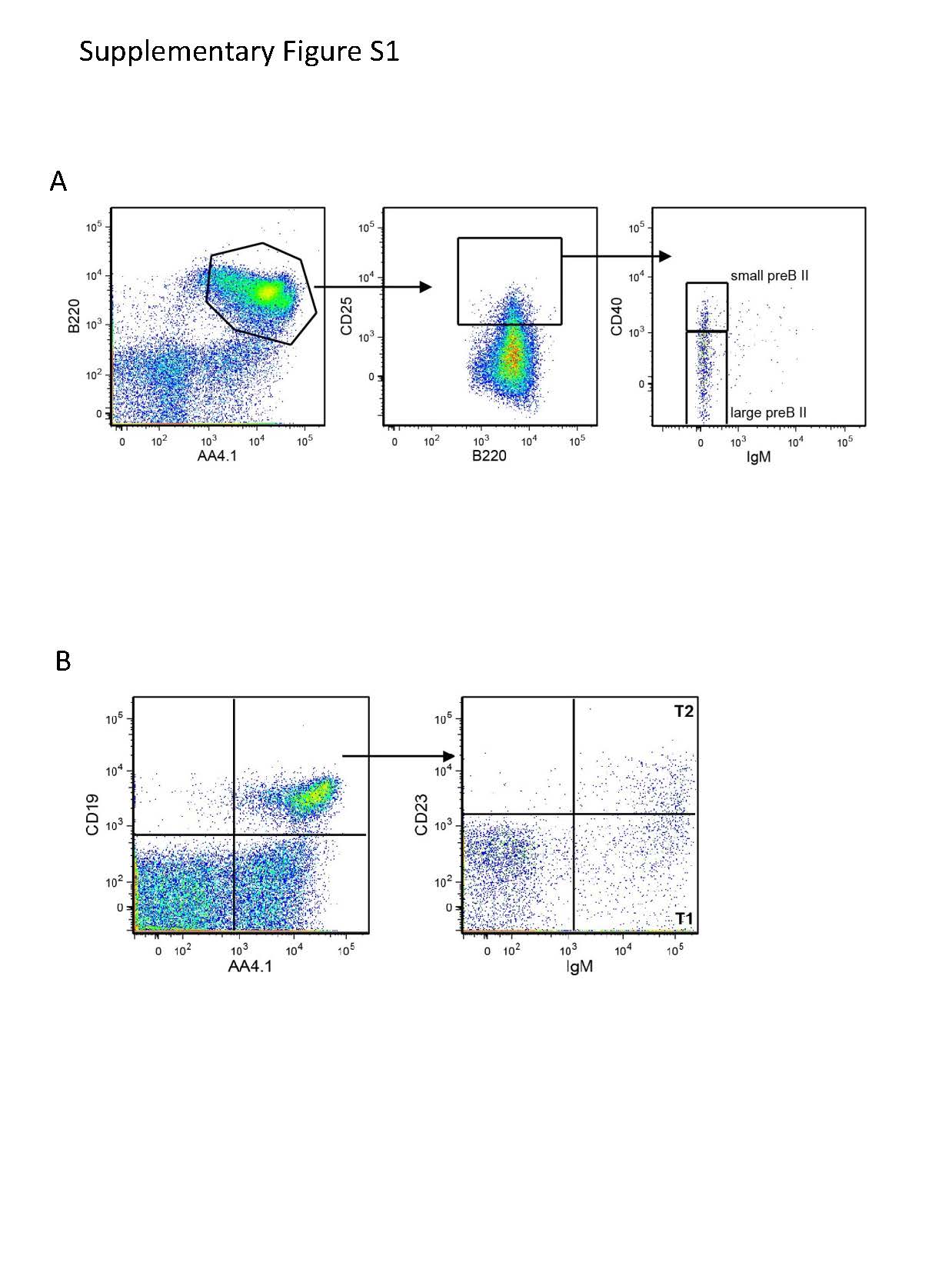

Supplement: Supplementary Figure S1 — Flow cytometric gating for large and small preB cells as well as for T1 and T2 transitional cells. (A) Shown is an example of staining of BM cells from a B-Indu-Rag1 mouse analyzed 6 days after induction. Cells were gated for lymphocytes using forward and sideward scatter. Doublets were excluded by applying FSC area and heights against each other. Dead cells were excluded by gating for DAPI-negative cells. Those cells were then gated for B220+AA4.1+ cells for immature B cell population as shown and finally for CD25 and CD40. IgM bearing cells were excluded. (B) Shown is a representative staining for BM cells of an induced Indu-BRag1 mouse. Gating was done as in A. Transitional B cells amongst B cell progenitors could be differentiated into T1 and T2 as CD23−IgM+ (T1) and CD23+IgM+ (T2). Similar gating was performed for all tissues analyzed. [file Image_1.JPEG]

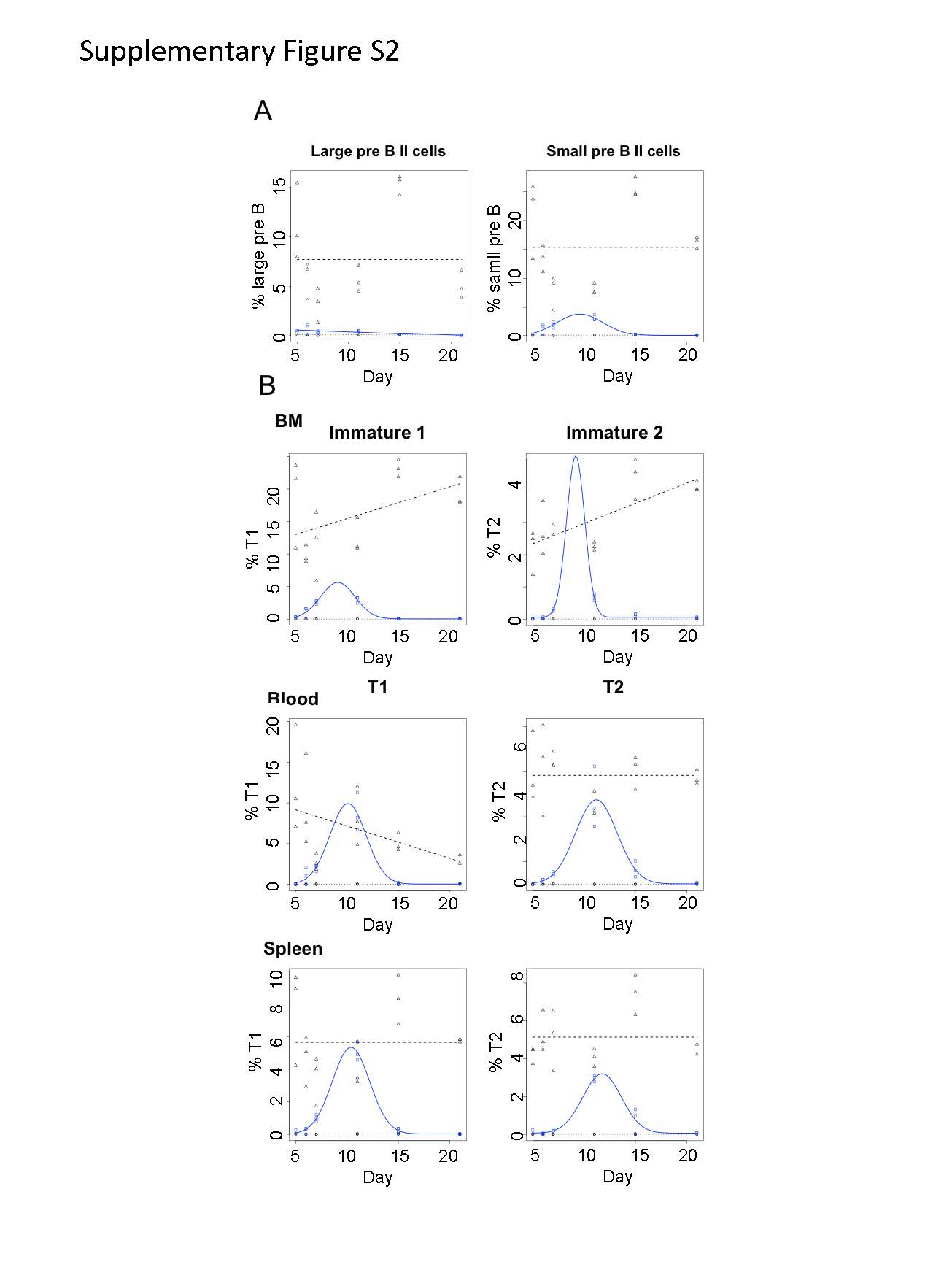

Supplement: Supplementary Figure S2 — Results of least squares fitting. In each case, a constant, a linear and a bell-shaped model were fitted. The lines shown correspond to the best model according to Akaike's information criterion. (A). Frequencies of indicated B cell developmental stages based on data from Figure 1A were used and fitted by least square statistics. (B). Data from Figure 1B were used and fitted by least square statistics. [file Image_2.JPEG]

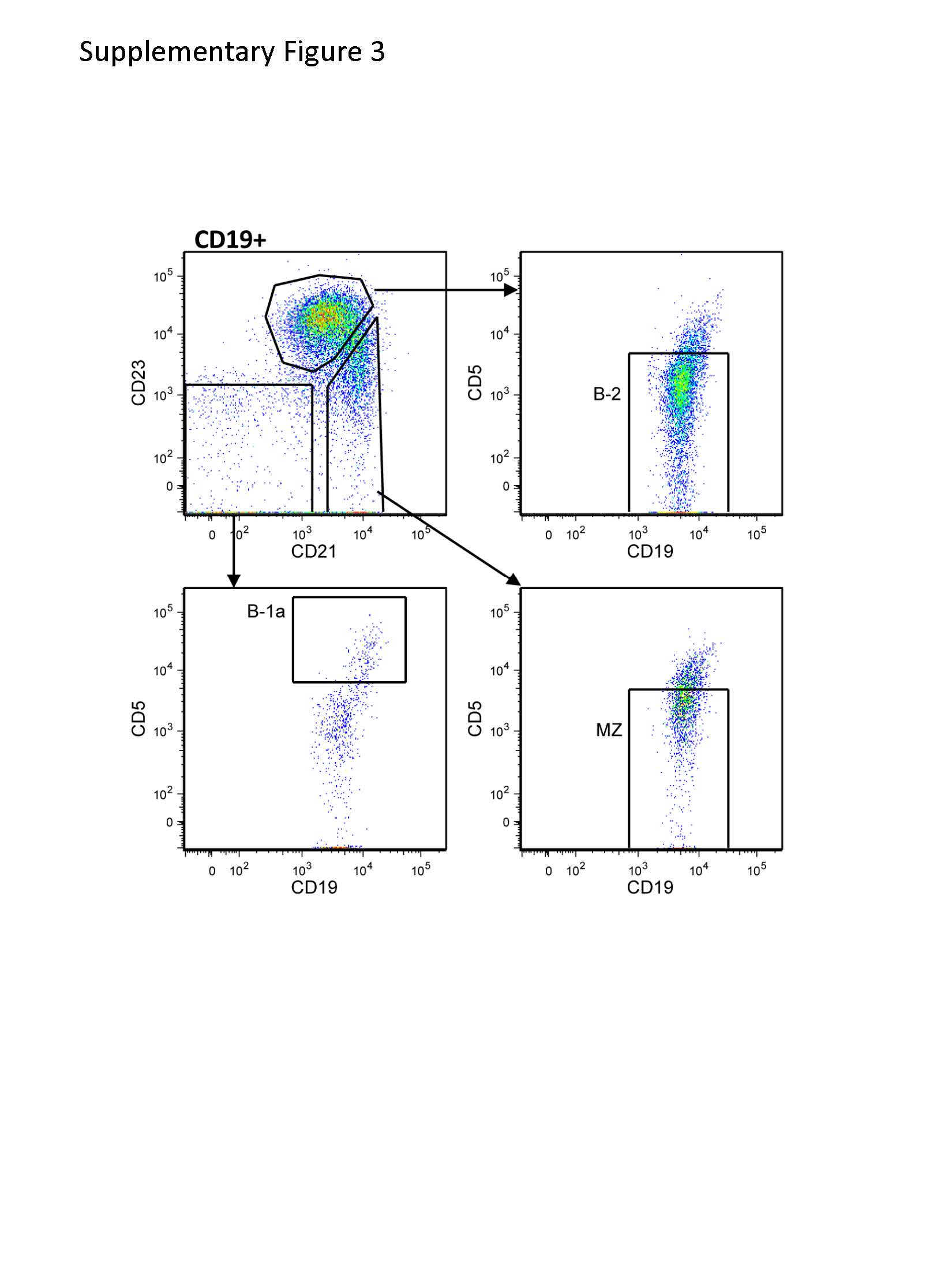

Supplement: Supplementary Figure S3 — Gating strategy for mature B cells. Shown is a representative staining for cells isolated from spleen of an induced B-Indu-Rag1 mouse. Cells were gated for lymphocytes first using forward and sideward scatter. Doublets were excluded by applying FSC area and heights against each other. Dead cells were excluded by gating for DAPI-negative cells. Those cells were then gated for CD19 and furthermore classified according to the figure and CD23, CD21, CD5 expression. [file Image_3.JPEG]

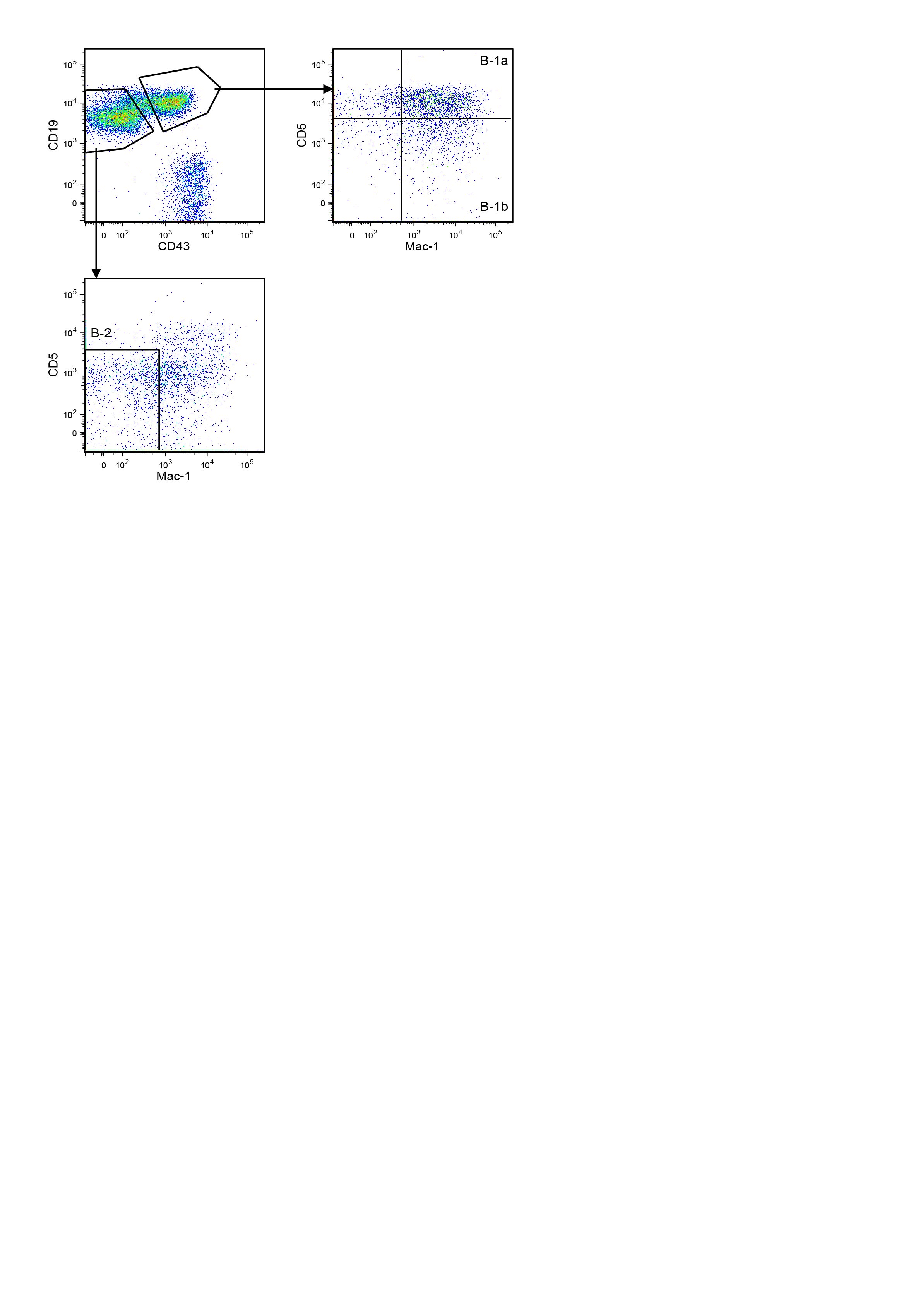

Supplement: Supplementary Figure S4 — Gating strategy for peritoneal mature B cell populations. Shown is a representative staining for cells isolated from peritoneal cavity of an induced B-Indu-Rag1 mouse. Cells were gated for lymphocytes first using forward and sideward scatter. Doublets were excluded by applying FSC area and heights against each other. Dead cells were excluded by gating for DAPI-negative cells. Cells were further subdivided based on their expression of CD19, CD43, Mac-1 (CD11b), and CD5. [file Image_4.jpg]

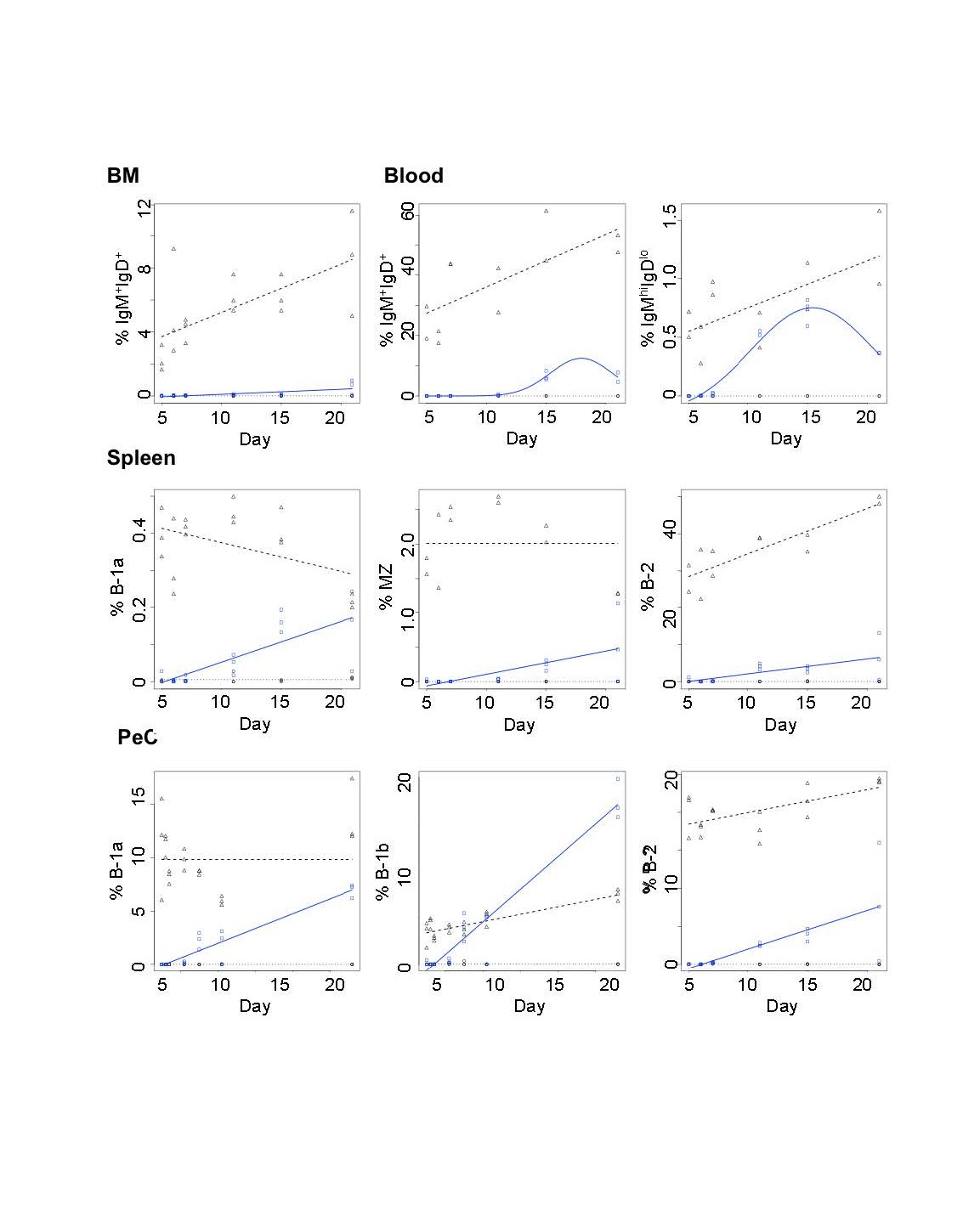

Supplement: Supplementary Figure S5 — Results of least squares fitting for mature B cells. Analysis was carried out like for Figure S2. Frequencies of indicated BCR+ B cell subsets based on data from Figure 2 were used and fitted by least square statistics. [file Image_5.jpeg]
